# Stable isotope analyses—A method to distinguish intensively farmed from wild frogs

**DOI:** 10.1002/ece3.2878

**Published:** 2017-03-14

**Authors:** Carolin Dittrich, Ulrich Struck, Mark‐Oliver Rödel

**Affiliations:** ^1^ Museum für NaturkundeLeibniz Institute for Evolution and Biodiversity ScienceBerlinGermany

**Keywords:** conservation tool, food production, hunting pressure, international trade, natural populations, wild‐caught

## Abstract

Consumption of frog legs is increasing worldwide, with potentially dramatic effects for ecosystems. More and more functioning frog farms are reported to exist. However, due to the lack of reliable methods to distinguish farmed from wild‐caught individuals, the origin of frogs in the international trade is often uncertain. Here, we present a new methodological approach to this problem. We investigated the isotopic composition of legally traded frog legs from suppliers in Vietnam and Indonesia. Muscle and bone tissue samples were examined for δ^15^N, δ^13^C, and δ^18^O stable isotope compositions, to elucidate the conditions under which the frogs grew up. We used DNA barcoding (16S rRNA) to verify species identities. We identified three traded species (*Hoplobatrachus rugulosus, Fejervarya cancrivora* and *Limnonectes macrodon*); species identities were partly deviating from package labeling. Isotopic values of δ^15^N and δ^18^O showed significant differences between species and country of origin. Based on low δ^15^N composition and generally little variation in stable isotope values, our results imply that frogs from Vietnam were indeed farmed. In contrast, the frogs from the Indonesian supplier likely grew up under natural conditions, indicated by higher δ^15^N values and stronger variability in the stable isotope composition. Our results indicate that stable isotope analyses seem to be a useful tool to distinguish between naturally growing and intensively farmed frogs. We believe that this method can be used to improve the control in the international trade of frog legs, as well as for other biological products, thus supporting farming activities and decreasing pressure on wild populations. However, we examined different species from different countries and had no access to samples of individuals with confirmed origin and living conditions. Therefore, we suggest improving this method further with individuals of known origin and history, preferably including samples of the respective nutritive bases.

## 1 Introduction

The international trade of frog legs encompassed five million tons of meat in 2007 (UN Statistics Division). In a period from 2003 to 2007, Indonesia and Vietnam were the main exporters with approximately four and 0.6 million tons per year, respectively. Recent overviews of the international frog leg trade have been published by Gratwicke et al. (2009), Warkentin, Bickford, Sodhi, and Bradshaw (2009), and Altherr, Goyenechea, and Schubert (2011). Unfortunately, there are no more recent export data available, as the specific declaration of amphibian meat has been removed from the trade database (UN Comtrade) in 2007 (Gerson, 2012). Now, frog meat is being included in “other meat and edible offal” (HS 0208.90). The low trade volume was given as the reason for deletion of the subheading (World Customs Organization, 2012).

Nevertheless, the demand for frog legs is increasing in many parts of the world, for two different reasons. First, poor nutritional condition and declining mammal and fish populations seem to trigger an increasing demand for alternative protein sources in many developing countries, especially in South‐East Asia and Africa (Mohneke, Onadeko, Hirschfeld, & Rödel, 2010; Neang, 2010). Second, frog legs are “rediscovered” as a delicacy or as a status symbol, in particular in the European Community and the United States (Altherr et al., 2011), but as well by a growing rich and middle‐class community in developing countries (see, e.g., the Goliath frog in Cameroon; Gonwouo & Rödel, 2008).

In 2002, the food and agriculture organization of the United Nations (FAO) estimated that 15% of the global market of frogs (all species) is supplied by aquaculture, indicating that the remaining 85% are still taken from wild populations. Respective over‐harvesting of these populations could lead to declining frog populations and an increase in pest organisms (Oza, 1990), as well as to other negative ecosystem consequences (Mohneke & Rödel, 2009). Apparently, the high demand for frog legs cannot be met by farming, even considering an increasing number of frog farms (Aabedi, Mirsaeed, & Khoshbakht, 2014; Helfrich, Neves, & Parkhurst, 2009; Moreira, Henriques, & Ferreira, 2013) and ongoing research to improve the yield (Ding, Lin, Fan, & Ji, 2015; Gui & Zhu, 2012; Martínez, Real, & Álvarez, 2004). So far quality standards for frog aquaculture do not exist (Nguyen, 2014). Most frog leg packages in Berlin supermarkets (this study) seem to stem from farming. Given the correctness of the above figures, this seemed rather unlikely.

Unfortunately, there is no easy/applicable method to determine if frog legs are farmed or wild‐caught. Thus, the percentage of frog legs with a nonfarming origin could be even higher than believed. Different attempts were made to shed light on the identity of traded species, but to know which species are involved does not necessarily allow conclusions if they were farmed or wild‐caught (Veith, Kosuch, Feldmann, Martens, & Seitz, 2000). Another promising attempt was measuring femoral bone density, where frogs from captivity show lighter bones (Yang, Huang, Xia, Xu, & Dahmer, 2011). However, this method is rather complex and difficult to standardize. We, therefore, searched for another option.

In forensic science, the use of stable isotopes is established to uncover the origin and living conditions of individuals (Bowen, Wassenaar, & Hobson, 2005; Meier‐Augenstein & Fraser, 2008; Retief, West, & Pfab, 2014). The stable isotope composition in muscle tissue, teeth, hair or bone can indicate, for example, conditions during growth, migration between sites or source and quality of diet (Hobson, 1999; Voigt et al., 2014). The relative food web position can be inferred by nitrogen isotope values (δ^15^N), as heavy isotopes get accumulated in the food chain (Fry, 1991; Minagawa & Wada, 1984). For each trophic level, the nitrogen isotopic ratio increases by approximately 3‰ (Fry, 2006). The carbon isotopes (δ^13^C) in tissue samples are markers for the source of energy and thus indicate broad habitat categories, like carbons produced by C3 (more forested and mid to higher latitude environments) or C4 plants (more open tropical grass dominated habitats), and are distinguishable by carbon isotopic compositions around −28‰ (C3) and −14‰ (C4; Fry, 2006), respectively. The oxygen isotope composition (δ^18^O) in bone carbonate samples is a marker for the geographic origin, due to spatial variation of isotopes in precipitation and thermal conditions, for example coastal lowlands show higher δ^18^O values, compared to continental mountains (Hobson, 1999).

The combination of multi‐element isotope signatures allows—to some extent—tracing the living conditions of an individual. We herein assume that the intraspecific variability in particular isotope values should be higher in populations growing under natural conditions (different food items, dietary sources and climatic conditions, depending on microhabitat, etc., Bearhop, Adams, Waldron, Fuller, & Macleod, 2004) than under intensive farming conditions (same food and water source, climatic conditions, etc.), as it has been shown experimentally in the Crocodile lizard *Shinisaurus crocodilurus* (van Schingen et al., 2016). Exact information on pelleted food components, used in frog farms, is scarce, but protein content should vary between 28% and 40% (Miles, Williams, & Hailey, 2004; Pariyanonth & Daorerk, 1994). Unfortunately, no information concerning the protein source is available (plant or animal, terrestrial, or marine), which could influence the isotopic composition (Schoeninger & DeNiro, 1984). Nevertheless, the protein content in arthropods, the dominant food sources of wild frogs (Wells, 2007), is usually higher (Xiaoming, Ying, Hong, & Zhiyong, 2010) than in pelleted food. Therefore, we expect enriched δ^15^N values if frogs were taken from the wild.

We herein present a possible approach to answer the question if a traded frog was intensively farmed, reared under natural‐like conditions or caught from the wild, by comparing isotopic values from tissue samples of legally traded frog legs. Our hypotheses were the following: (1) the nitrogen isotopic signal from intensively farmed individuals should be lower than expected from naturally growing populations (lower protein source food/trophic level) and (2) the variability in isotopic values should be lower in intensively farmed individuals (same conditions for all individuals), compared to those growing under natural conditions.

## 2 Material and methods

### 2.1 Origin of frog legs

Seven packages, one kg each, of deep frozen frog legs were obtained from supermarkets in Berlin, Germany. According to the package labeling, the frog legs originated from two suppliers in Vietnam and one from Indonesia (Table 1). The statement of origin on the Vietnam packages was labeled with “farmed.” Based on the EU code on the packages, we could identify the geographic locations of both Vietnamese suppliers being in the Mekong delta, thus matching other information about the main farming activities in the country (Quoc, 2012). On the Indonesian packages, there was no such statement and we could only trace back to a leading Indonesian company trading frozen food. A former study on frog leg trade in Indonesia revealed that native frogs are not farmed in Indonesia (Kusrini & Alford, 2006). Unfortunately, we had no access to frog tissue from neither wild Vietnamese nor Indonesian populations, or directly from frog farms. Our study thus was limited to frog legs which are freely and legally traded, and consequently, indirect evidence and comparisons to literature data. The only suppliers offering frog legs in Berlin supermarkets were from Vietnam and Indonesia; it was thus impossible to include frog legs from other suppliers and countries.

**Table 1:** Origin and quantity of frog legs tested in this study. Given is the country from where the frog legs were shipped, the statement of origin and the species name given on the packages, package number (charge no.), the total weight (kg) and number (*n*) of frog legs per package, as well as the date when the package was packed

Country	Statement of origin	Species	Charge no.	Quantity	Date
Vietnam (1)	Farmed in Vietnam	*Hoplobatrachus rugulosus*	VN/364/IV/225	3 kg *n* = 46	22.10.2013
Vietnam (2)	Farmed in Vietnam	*Hoplobatrachus rugulosus*	VN/028/IV/001	2 kg *n* = 34	13.01.2014
Indonesia	Indonesia	*Rana macrodon*	095.13.B	2 kg *n* = 45	19.05.2013

### 2.2 DNA barcoding

In order to test if the species declaration on the packages were correct, we checked the frog legs identity by applying a DNA barcoding approach (compare Vences, Thomas, Van der Meijden, Chiari, & Vieites, 2005). The knowledge of species identity was necessary for our study as different species might differ in their stable isotope compositions (Kupfer, Langel, Scheu, Himstedt, & Maraun, 2006; Verburg, Kilham, Pringle, Lips, & Drake, 2007). A small piece of muscle tissue from right thighs (thus avoiding double testing of the same individual) was used to gain mitochondrial DNA (16S rRNA). We used 16S rRNA, as for most internationally traded frog species these sequences are available in GenBank (Veith et al., 2000). All sequencing was done by “Services in Molecular Biology GmbH” (SMB, Berlin). The primers used for PCR and sequencing were 16SA‐L and 16SB‐H for amphibians (16SA‐L, 5‐CGC CTG TTT ATC AAA AAC AT‐3′; 16SB‐H, 5′‐CCG GTC TGA ACT CAG ATC ACG T‐3′.). We analyzed approximately 500 bp of 16S rRNA of 97 specimens. Sequences were aligned with CodonCode Aligner software (v. 5.0.2, CodonCode Corporation, Dedham, MA, USA) and compared to samples from GenBank. Uncorrected *p*‐distances were calculated using MEGA6 (Tamura, Stecher, Peterson, Filipski, & Kumar, 2013). All sequences derived from that study have been uploaded to GenBank (accession numbers in Appendix).

### 2.3 Isotope composition

We used muscle tissue samples from the right thighs (*n* = 125) to determine nitrogen and carbon isotope composition (δ^13^C and δ^15^N), which indicates trophic level in the food chain and the energy source of the consumed food; and oxygen and carbon isotope composition (δ^13^C and δ^18^O) from femoral bones, indicating geographic origin and environmental variability (water source, microhabitat). Muscle tissue was dried at 60°C in a drying chamber for a minimum of 24 hr.

The nitrogen and carbon stable isotope analysis and concentration measurements were performed simultaneously with a THERMO/Finnigan MAT V isotope ratio mass spectrometer (Thermo Finnigan, Bremen, Germany), coupled to a THERMO Flash EA 1112 elemental analyzer via a THERMO/Finnigan Conflo IV interface, in the stable isotope laboratory of the Museum für Naturkunde, Berlin. Stable isotope ratios are expressed in the conventional delta‐notation (δ^15^N, δ^13^C), relative to atmospheric nitrogen (Mariotti, 1983) and Vienna PeeDee Belemnite standard (VPDB). Standard deviation for repeated measurements of laboratory standard material (peptone) was generally better than 0.15 per mill (‰) for nitrogen and carbon, respectively.

The femoral bones for the oxygen measurements were mechanically cleaned from tissue, bone marrow, and joint capsules. The remaining bone was powdered in a porcelain mortar. We added 1 ml of 4% sodium hypochlorite (NaOCl) for 2–3 days to remove remaining organic matter and changed NaOCl after 1 day (Balasse, Bocherens, & Mariotti, 1999). After removal of the NaOCl, we washed the bone powder until neutrality (minimum five times with distilled water; Balasse et al., 1999). Afterward, we added 1 ml of acetic acid (C_2_H_4_O_2,_ 0.1 mol/L) for 4 hr, during which the samples were constantly moved on a rotator. After removal of acetic acid, we washed the powder until neutrality (minimum five times with distilled water) and dried it in a dryer chamber at 40°C (minimum 12 hr) to remove all water, which otherwise could distort oxygen values (Balasse et al., 1999).

For oxygen and carbon isotope measurements of bone samples, approximately 1–4 mg of sample material was put into a clean 10‐ml glass vial (exetainer^®^, LABCO Limited, Lampeter, UK). After sealing the exetainer^®^ with a septum cap (caps and septa for LABCO exetainer^®^ 438b), the remaining air was removed by flushing the exetainer^®^ with He (4.6, purity ≥99.996%) for 6 min, at a flow rate of 100 ml per minute. After flushing, approximately 30 μl of anhydrous phosphoric acid (H_3_PO_4_, 100%) was injected through the septum into the sealed exetainer^®^, using a disposable syringe. After 1.5 hr of reaction time at 50°C, the sample was ready for isotope measurement.

The oxygen and carbon isotopic composition in the CO_2_ in the headspace was measured using a Thermo Finnigan GASBENCH II coupled online with a Thermo Finnigan delta V isotope ratio mass spectrometer. Reference gas was pure CO_2_ (4.5, purity ≥99.995%) from a cylinder calibrated against the VPDB standard, using reference materials (NBS 18, NBS 19) from the International Atomic Energy Association (IAEA). Isotope values are given in the conventional delta‐notation (δ^18^O, δ^13^C) in per mil (‰) versus Vienna Standard Mean Ocean Water (VSMOW) and VPDB, respectively. Reproducibility of replicate measurements of laboratory standards (limestone) was generally better than 0.10‰ (one standard deviation).

All data showed non‐normal distribution (Shapiro test) and heterogenic variances (Bartlett test). Therefore, we used Kruskal–Wallis nonparametric test to check for significant differences between the individual packages and respective species. Afterward, we conducted pairwise group comparisons using Mann–Whitney *U*‐tests, with a correction for multiple comparisons of *p*‐values (correction method = “false discovery rate,” Benjamini & Hochberg, 1995). All statistical analyses were conducted with statistical software R (R Core Team, 2015; base package); we used the package ggplot2 (Wickham, 2009) for visualization.

The measured isotopic values were compared to values derived from natural populations if available in literature, for example, of *Hoplobatrachus rugulosus* (δ^15^N = 8‰, Kupfer et al., 2006).

### 2.4 Comparative data

For comparative isotopic data, we screened the literature for amphibian species reflecting different trophic systems/classifications, body sizes and ecological niches (leaf litter frogs, tree frogs, terrestrial or aquatic species), because all these factors could influence stable isotope composition (Bearhop et al., 2004; Fry, 2006; Jefferson & Russel 2008). As for our approach the variability of values from adult frogs was important we only considered data from adult frogs with more than one individual per species included.

## 3 Results

### 3.1 DNA barcoding

**Figure 1:**
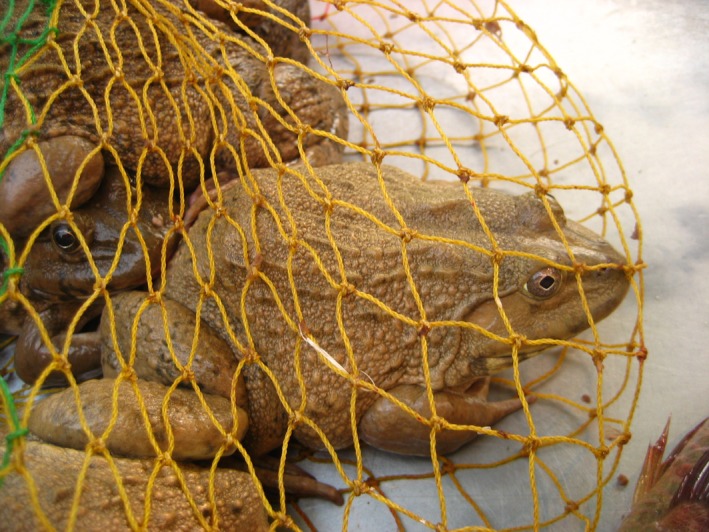
*Hoplobatrachus rugulosus* sold alive on a local market in Bang Phra subdistrict, Chon Buri province, Thailand. Photographer: Carolin Dittrich

To identify the traded species, we compared our 16S rRNA sequences with sequences deposited in GenBank (Appendix). The DNA barcoding revealed that all tested animals from Vietnam (*n* = 49) belonged to the species *H. rugulosus* (Wiegmann, 1834; Figure 1)*,* as declared on the packaging (uncorrected *p*‐distance: 0%, all samples being absolutely identical).

The frog legs from the Indonesian distributer were all labeled *Rana* (=*Limnonectes*) *macrodon*. However, we identified two species in the respective packages; 13 individuals of *Limnonectes macrodon* (Duméril and Bibron, 1841) (uncorrected *p*‐distance: mean = 0.34%, range = 0.20%–0.50%), listed as Vulnerable (IUCN RedList; Iskander, Mumpuni, Das, & Ohler, 2004), and 32 *Fejervarya cancrivora* (Gravenhorst, 1829) individuals (uncorrected *p*‐distance: mean = 0.20%, range = 0.00%–0.51%) listed as Least Concern (IUCN RedList; Zhigang et al., 2004).

### 3.2 Stable isotope composition

**Figure 2:**
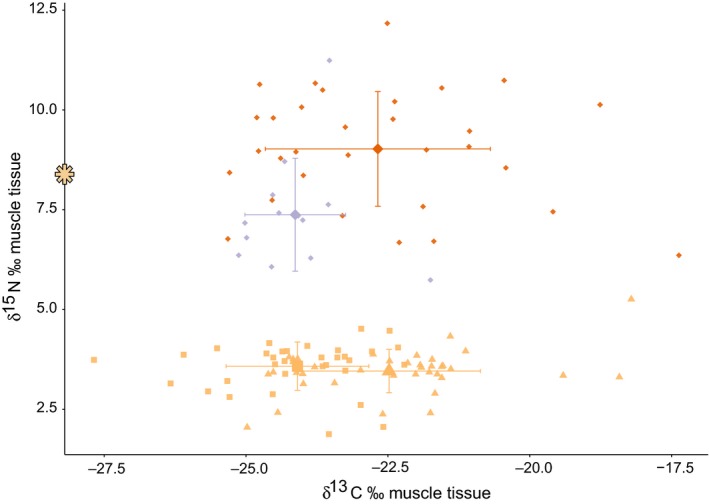
Nitrogen (δ^15^N) and carbon (δ^13^C) isotopic composition of leg muscles from three frog species: *Fejervarya cancrivora* (orange diamonds, *n* = 32), *Limnonectes macrodon* (violet diamonds, *n* = 13), and *Hoplobatrachus rugulosus* (Vietnam 1: yellow triangles, *n* = 46; Vietnam 2: yellow squares, *n* = 34). As comparison, the star on the *y*‐axis represents a nitrogen isotope value from a natural population of *H. rugulosus* (Kupfer et al., 2006). Displayed are the values of dried muscle tissue for single samples, in ‰. The bigger symbols depict average values per species and packages (Vietnam, compare text) with error bars (standard deviations); compare Table 2

The isotopic composition of the muscle tissue differed largely between samples. The samples from Indonesia showed average δ^15^N values around 7‰–9‰, the two species having specific values, with *F. cancrivora* showing higher values (mean ± *SD* = 9.0‰ ± 1.4‰, range = 6.6‰–12.2‰) than *L. macrodon* (mean ± *SD* = 7.4‰ ± 1.4‰, range = 5.7‰–11.7‰). The range of nitrogen isotopic values was high in both species (Table 2, Figure 2). The samples from Vietnam showed lower and less variable δ^15^N values around mean ± *SD* = 3.5‰ ± 0.6‰, where Vietnam 1 ranged from 2.0‰ to 5.3‰ and Vietnam 2 from 1.9‰ to 4.5‰ (Table 2, Figure 2). Compared to Vietnamese samples, Indonesian frog legs showed enriched δ^15^N values and exhibited a three times higher standard deviation. When comparing our isotopic values to literature data, the δ^15^N values for the Vietnamese *H. rugulosus* were only about half as high and showed little variation compared to values derived from natural populations (δ^15^N mean = 8‰ Kupfer et al., 2006). In comparison with nitrogen, carbon values varied more strongly (Table 2, Figure 2).

**Table 2:** Summary of isotopic values (mean and standard deviation; ‰) for bone and muscle tissue of *Fejervarya cancrivora* (*n* = 32), *Limnonectes macrodon* (*n* = 13), and *Hoplobatrachus rugulosus* (Vietnam 1, *n* = 46, Vietnam 2, *n* = 34), given is also leg length (mean and standard deviation; mm), values being an indication that all frogs were fully grown

			Bone tissue		Muscle tissue	
Species	Origin	Leg lengthmm	δ^18^O	δ^13^C	δ^15^N	δ^13^C
*F. cancrivora*	Indonesia	86.4 ± 4.3	24.4 ± 2.0	−13.9 ± 1.6	9.0 ± 1.4	−22.7 ± 2.0
*L. macrodon*	Indonesia	94.7 ± 6.5	24.1 ± 2.3	−14.7 ± 0.9	7.4 ± 1.4	−24.1 ± 0.9
*H. rugulosus*	Vietnam 1	79.7 ± 6.3	20.8 ± 1.1	−15.6 ± 0.5	3.5 ± 0.5	−22.5 ± 1.6
	Vietnam 2	81.1 ± 5.6	20.5 ± 1.9	−15.8 ± 1.1	3.6 ± 0.6	−24.1 ± 1.3

We compared four sample groups, based on the three species involved and their respective suppliers (*L. macrodon* from Indonesia, *F. cancrivora* from Indonesia, *H. rugulosus* from Vietnam 1 and *H. rugulosus* from Vietnam 2). The Kruskal–Wallis test comparisons between groups revealed significant differences in nitrogen (Chi^2^ = 85.25, *df* = 3, *p *<* *.001) and carbon composition (Chi^2^ = 22.13, *df* = 3, *p *<* *.001). The detailed pairwise comparisons showed significant differences in nitrogen and carbon composition between all pairs (Mann–Whitney *U*‐test, *p *<* *.05).

**Figure 3:**
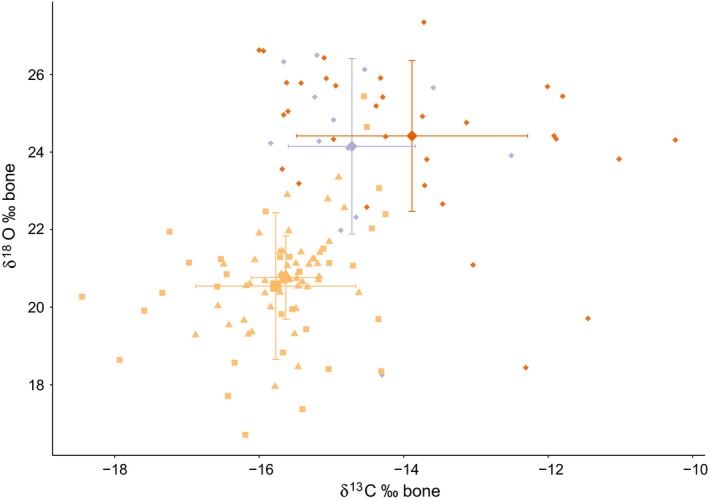
Oxygen (δ^18^O) and carbon (δ^13^C) isotopic composition of grinded bone powder for three frog species: *Fejervarya cancrivora* (orange diamonds, *n* = 32), *Limnonectes macrodon* (violet diamonds, *n* = 13), and *Hoplobatrachus rugulosus* (Vietnam 1: yellow triangles, *n* = 46; Vietnam 2: yellow squares, *n* = 34). Displayed are the values of bone powder for single samples, in ‰. The bigger symbols depict average values per species and packages (Vietnam, compare text) with error bars (standard deviations); compare Table 2

The δ^18^O and δ^13^C values from Vietnam bone samples were lower, compared to Indonesian samples (Table 2, Figure 3). The between‐group comparison revealed significant differences in oxygen (Chi^2^ = 58.71, *df* = 3, *p *<* *.001) and carbon isotopic composition (Chi^2^ = 38.21, *df* = 3, *p *<* *.001). The detailed pairwise comparisons showed significant differences in oxygen and carbon isotopic composition between the origin of the frog legs (Indonesia vs Vietnam; Mann–Whitney *U*‐test, *p *<* *.05).

### 3.3 Comparative data

We traced isotopic measurements for a variety of amphibian species from different continents. These data show that wild populations exhibit δ^15^N values above a minimum of 4‰, the majority of species showing values above 6‰ (Table 3). These measurements indicate that our samples from Vietnam were indeed depleted in their amount of δ^15^N and thus differed from what is usually observed in wild populations.

**Table 3:** Summary of isotopic values of frog tissue samples (δ^15^N ‰, δ^13^C ‰) from wild populations and respective sample sizes (*n*) from Middle America (1 =  Verburg et al., 2007), South‐East Asia (2 = Kupfer et al., 2006), New Zealand (3 = Najera‐Hillman, Alfaro, Breen, & O'Shea, 2009) and North America (4 = Jefferson & Russel 2008)

Species	δ^15^N ‰	δ^13^C ‰	*n*	Source isotope	Size of species
*Hyloscirtus palmeri* (Boulenger, 1908)	4.39 ± 0.02	−25.0 ± 0.39	2	1	36–50 mm^a^
*Lithobates warszewitschii* (Schmidt, 1857)	6.29 ± 0.64	−25.4 ± 0.26	3	1	37–63 mm^a^
*Rhaebo haematiticus* Cope, 1862	8.05 ± 0.21	−24.4 ± 0.08	3	1	42–80 mm^a^
*Hyloscirtus colymba* (Dunn, 1931)	4.58 ± 0.05	−25.1 ± 0.60	3	1	31–39 mm^a^
*Silverstoneia flotator* (Dunn, 1931)	5.11 ± 0.92	−24.2 ± 2.17	3	1	14–18 mm^a^
*Silverstoneia nubicola* (Dunn, 1924)	5.77 ± 0.33	−26.8 ± 2.09	3	1	15–21 mm^b^
*Espadarana prosoblepon* (Boettger, 1892)	5.27 ± 0.15	−25.1 ± 1.79	3	1	21–31 mm^c^
*Colostethus panamensis* (Dunn, 1933)	5.99 ± 0.77	−26.0 ± 1.07	3	1	27–28 mm^d^
*Hoplobatrachus rugulosus* (Wiegmann, 1834)	8.00 ± 0.20	–	2	2	72–128 mm^e^
*Occidozyga lima* (Gravenhorst, 1829)	7.50 ± 0.50	–	3	2	39–40 mm^f^
*Occidozyga martensii* (Peters, 1867)	6.00 ± 0.50	–	2	2	19–33 mm^g^
*Microhyla pulchra* (Hallowell, 1861)	6.60 ± 1.30	–	5	2	23–37 mm^h^
*Microhyla ornata* (Dumeril and Bibron, 1841)	6.70 ± 1.30	–	2	2	13–26 mm^h^
*Microhyla butleri* Boulenger, 1900	6.70 ± 0.70	–	3	2	20–26 mm^h^
*Glyphoglossus mollossus* Günther, 1869	6.30 ± 0.20	–	3	2	41–94 mm^i^
*Glyphoglossus guttulata* (Blyth, 1856)	7.00 ± 0.30	–	3	2	34–50 mm^f^
*Leiopelma hochstetteri* Fitzinger, 1861	4.48 ± 0.17	−25.4 ± 0.38	3	3	20–46 mm^j^
*Lithobates clamitans* (Latreille, 1801) natural site	5.10 ± 0.30	−26.2 ± 0.10	7	4	38–80 mm^k^
*Lithobates clamitans* (Latreille, 1801) natural site	4.60 ± 0.30	−27.2 ± 0.90	8	4	38–80 mm^k^
*Lithobates clamitans* (Latreille, 1801) golf course site	9.15 ± 0.40	−28.1 ± 0.70	9	4	38–80 mm^k^
*Lithobates clamitans* (Latreille, 1801) agriculture site	11.40 ± 0.50	−26.1 ± 0.30	9	4	38–80 mm^k^

Sizes of species according to ^a^Savage (2002), ^b^Grant and Myers (2013), ^c^Cisneros‐Herida & McDiarmid (2007), ^d^Grant (2004), ^e^Zhi‐hua and Xiang (2005), ^f^Manthey and Grossmann (1997), ^g^Tran (2013), ^h^Poyarkov et al. (2014), ^i^Laojumpon, Suteethorn, and Lauprasert (2012), ^j^Green and Tessier (1990), ^k^Gorman and Haas (2011). Taxonomy updated according to Frost (2016). Dashes indicate that no data was available.

## 4 Discussion

We aimed to find a method to easily and reliably distinguish intensively farmed frogs from those growing under natural conditions. Herein, we present evidence that stable isotope analyses may serve as such a useful tool. Currently traded frogs apparently stem from both origins. The isotopic signals of the Vietnamese individuals most likely indicate intensive farming conditions, whereas the individuals from Indonesia seem to have originated from wild populations or farming conditions which are close to natural ones.

Although the frog species from both countries differed in their isotopic composition, their biology is very much alike; all being large‐sized ranids with similar prey spectrum (Almeria & Nuñeza, 2013; Elliott & Karunakaran, 1974; Kusrini, 2005; Zhi‐hua & Xiang, 2005). When comparing our isotopic values to literature data, the values for *L. macrodon* and *F. cancrivora* from Indonesia were in the range of other larger frogs with a broad spectrum of food items (Table 3). In contrast, the δ^15^N values of the Vietnamese *H. rugulosus* were only about half as high with little variation (Table 2); and thus below all wild population values which have been reported from a variety of different anuran species from different continents and ecological niches (Table 3). As we had no access to food samples to correct for the trophic baseline position (Post, 2002), and neither specific fractionation nor discrimination factors for the respective species are known (Cloyed, Newsome, & Eason, 2015; Vander Zanden & Rasmussen, 2001), we cannot make quantitative statements on food item composition. Still, based on the qualitative data (Layman et al., 2012), we can safely assume that our Vietnamese data are reflecting a low trophic level (Cabana & Rasmussen, 1994). The higher δ^15^N values, observed in single individuals, might be a result of cannibalism within a farming environment (Helfrich et al., 2009).

However, not only the δ^15^N values alone suggest a restricted/noncarnivorous diet of Vietnamese frogs and thus a farming origin; different growing conditions between Vietnamese and Indonesian frogs were also indicted by the differences of standard deviations between the isotopic values of our samples. Unfortunately, our standard deviations are not comparable with literature data, as the latter usually comprise low sample sizes (Table 3). Data from larval anurans, however, indicate that under natural conditions, variation of isotopic values increases with larger sample size (San Sebastián, Navarro, Llorente, & Richter‐Boix, 2015). The low variability in our Vietnamese frog legs, based on comparatively large sample size, therefore supports our assumption of an intensive farming environment, with restricted diet and common environment. Additionally, a study on crocodile lizards revealed that captive breed lizards displayed a smaller trophic niche width than wild‐caught animals, which was explained by a more restrictive diet (van Schingen et al. 2016). A final argument for the farming origin of Vietnamese frogs was the absence of any genetic variation of the tested samples.

However, in light of economic cost–benefit evaluation the provisioning of frogs with live prey is too expensive and time‐consuming to make a frog farm profitable (Aabedi et al., 2014; Helfrich et al., 2009; Miles et al., 2004; Quoc, 2012; Real, Martínez, & Álvarez, 2005). After all, we cannot give conclusive proof that the frogs from Indonesia are caught from the wild. However, former studies support this finding by stating that native frogs are not farmed in Indonesia (Kusrini & Alford, 2006). All available information from Indonesia suggests that only *Lithobates catesbeianus* (Shaw, 1802) is intensively farmed in that country (e.g., http://indoprimabullfrog.blogspot.de/; Kusrini, 2014). However, apart from natural populations another option of frog origin in Indonesia could be free‐ranged farming, where rice paddies are modified to support natural frog and fish populations to increase their abundance for commercialization (Shangkun, Mingyu, & Yong, 2015). These attempts to increase ecosystem services of rice paddies are also under investigation in other parts of Asia (Naito, Yamasaki, Imanishi, Natuhara, & Morimoto, 2012; Natuhara, 2013). In that natural farming system, frogs should show high nitrogen values and high variation as well, due to natural food variability.

Therefore, to validate the method, feeding trials and comparison of frogs with known origin from natural, seminatural, and farm populations should be conducted.

The range of the other isotopic values (δ^13^C in the tissue and δ^18^O from the bone) matches what could be expected from literature data. In South‐East Asia, rice is the main agricultural crop, and most of our frogs can be found in or close to rice fields (Kusrini, 2005). We measured δ^13^C values which are in the range of a C3 plant‐based diet (Fry, 2006) and therefore assume a C3 environment. As we could not get access to food samples used in commercial frog farming, we were unable to establish a baseline for comparison. The oxygen isotopes from the bone samples comprise an origin signal and indeed differed between the locations of the suppliers in Indonesia and Vietnam. Based on values of the global network of isotopes in precipitation (GNIP; IAEA/WMO 2015), we expected low oxygen values in the Vietnamese samples. Lower values of stabile oxygen isotopes are known from the Mekong Delta. Our results confirmed that expectation. We are hence confident that our results are reliable and reproducible.

The DNA barcoding mostly confirmed the declaration of species, but we identified one further species, *Fejervarya cancrivora*, in the Indonesian packages. This species is listed as of Least Concern (Zhigang et al., 2004) and is very similar to *L. macrodon*. Both are large ranids, which makes it hard to distinguish them after processing (Veith et al., 2000). The frogs identified herein are consistent with knowledge about South‐East Asian species traded for international food consumption (Altherr et al., 2011; Kusrini & Alford, 2006; Truong, 2000; Veith et al., 2000).

For thousands of years, frogs have been used by humans as food, in medicine, for cultural reasons or in science (Das, 2012; Tyler, Wassersug, & Smith, 2007). However, more recently their usage started to exceed sustainable levels (Altherr et al., 2011; Kusrini & Alford, 2006; Mohneke et al., 2010; Truong, 2000). This could lead to negative and unwelcome consequences for the respective ecosystems and men (Bowatte, Perera, Senevirathne, Meegaskumbura, & Meegaskumbura, 2013; Cortés‐Gomez, Ruiz‐Agudelo, Valencia‐Aguilar, & Ladle, 2015; Hocking & Babbitt, 2014). Therefore, the control of the origin and rearing conditions of frogs, available in the international food trade, seems advisable. Our approach of using isotopic composition analyses seems to be a useful tool for such control, based on legislation and conservation issues (custom control of correct declaration, monitoring of frogs’ origin, etc.), thereby supporting farming activities and decreasing pressure on wild populations. However, it likewise should be kept in mind that so far no international quality standard exists for frog aquaculture; and frog farms, at least in Vietnam, are not regulated (Nguyen, 2014). Hence, frog legs are not controlled by state or health authorities and no sanitary permits will be issued. Therefore, they could be potential vectors for amphibian diseases (e.g., *Bd* or *Bsal*) and could negatively affect wild amphibian populations via the international trade (Auliya et al., 2016; Gilbert et al., 2012; Gratwicke et al., 2009).

## Author contribution

M.‐O. R. and U.S. designed the work. C.D. performed data collection, analyzed data, and drafted the main part of the paper. U.S. performed stable isotope measurements. All authors discussed the results and implications and critically revised the manuscript. All authors gave their final approval of the current version to be published.

## Data accessibility

Stable isotope values for single frog legs are provided in Figures 2 and 3. All 16S rRNA sequences are archived at GenBank database, and accession numbers are given in 1.

## Conflict of interest

None declared.

## Appendix 1 Barcoding results of commercially traded frog legs from Vietnam and Indonesia. Given are species names, the GenBank accession numbers we used for comparison, the GenBank accession numbers of sequences generated in this study and the uncorrected *p*‐distances of the new sequences to the GenBank data (based on the 500 bp of the investigated part of the 16S rRNA gene)

### 1.1

**Table:**

Species	GenBank	New sequences	*p*‐distance in % (mean; range; number of individual comparisons)
*Hoplobatrachus rugulosus*	AB636617	KY609271–KY609321	0; 0–0; 49
*Limnonectes macrodon*	U66133	KY609258–KY609270	0.34; 0.20–0.50; 13
*Fejervarya cancrivora*	AB444684	KY609226–KY609239	0.20; 0.00–0.51; 32
